# A Review of Factors Influencing the Banking of Collected Umbilical Cord Blood Units

**DOI:** 10.1155/2013/463031

**Published:** 2013-02-25

**Authors:** David Allan, Tanya Petraszko, Heidi Elmoazzen, Susan Smith

**Affiliations:** ^1^Department of Medicine, Division of Hematology, University of Ottawa and Ottawa Hospital Research Institute, 501 Smyth Road, Box 701, Ottawa, ON, Canada K1H 8L6; ^2^Canadian Blood Services, BC & Yukon Centre, 4750 Oak Street, Vancouver, BC, Canada V6H 2N9; ^3^OneMatch, Canadian Blood Services, 1800 Alta Vista Drive, Ottawa, ON, Canada K1G 4J5

## Abstract

Umbilical cord blood banking efforts have increased dramatically in the past two decades in response to increasing demand for alternative sources of blood stem cells to support patients requiring hematopoietic stem cell transplantation. Transplant centres have accumulated increasing expertise in their understanding of umbilical cord blood characteristics that are associated with improved outcome following transplantation. These characteristics and factors can assist transplant centres in selecting cord blood units from the worldwide inventory of banked units. Umbilical cord blood banks, therefore, need to remain agile in adjusting the inventory of the banks to address shifts or changes in the needs of transplant centres. Public umbilical cord blood banks face the challenge of building inventory while managing limited resources and are faced with decisions regarding which units can be stored and which units that have been collected should be discarded or used for other endeavours such as research. To this end, we sought to review parameters influencing the decision to bank a collected cord blood unit. In this paper, we will address parameters associated with graft potency and address other factors that guide the decision to bank collected units.

## 1. Measures of Graft Adequacy in Cord Blood Transplantation: Implications for Unit Selection and Banking

Although umbilical cord blood is highly enriched for hematopoietic stem cells and progenitors, more widespread use in adult transplant recipients remains limited by the total dose of available cells. Consequently, recovery of cell counts after transplantation can be delayed and primary graft failure remains as high as 20% for single unit umbilical cord blood transplants in recent reports [1–3]. As transplant centres increasingly rely on alternative sources of blood-forming stem cells, the efforts of cord blood banks to collect and store units with greater capacity to achieve timely engraftment will be essential for continued improvements in patient outcomes. 

Measures of graft potency are surrogate markers associated with time to engraftment following hematopoietic stem cell transplantation. Several clinically relevant measures have emerged but none have been studied in randomized controlled trials. Retrospective observational cohort studies, experimental transplant research, and practical measures in common use have been identified as parameters related to successful and timely engraftment following umbilical cord blood transplantation. Hematopoietic engraftment is typically defined as the time after transplantation when the blood counts have recovered into a safe range and when patients no longer require transfusion support. Platelet engraftment is typically defined as the number of days after the day of stem cell infusion until platelets are >20 × 10^9^/L and/or >50 × 10^9^/L without platelet transfusion in the preceding 48 h. Neutrophil recovery is typically defined as the number of days until neutrophils are >0.5 × 10^9^/L and/or >1.0 × 10^9^/L [4]. These definitions are more challenging to apply following some reduced intensity conditioning regimens and molecular methods are often used to confirm the cells are donor in origin. Delayed recovery of platelets and neutrophils, however, is associated with increased risk of serious bleeding and/or serious infection with an associated increased risk of death [5]. 

Parameters that have been associated with graft adequacy are described below followed by a brief description regarding their clinical utility. In most cases, there are reports that support the relationship between a particular parameter and rates of hematopoietic engraftment or reduced rates of graft failure but for many cord blood units, the corresponding parameters of individual units may point in opposite directions with regard to graft potency. Optimal strategies, therefore, to guide public cord blood banks in the selection of collected units for banking remains an area of active discussion [6]. 

### 1.1. Total Volume Collected

This is a very simple but reliable measure with small interobserver variability. For this reason, volume remains an important and cost-effective parameter to estimate the blood-forming potential of cord blood units. Volume collected is most useful in the setting of making rapid decisions of whether banks should invest in the processing and analysis of collected units. Volume collected correlates well with hematopoietic measures of unit quality such as total nucleated cell count and CD34^+^ cell number [7, 8] and can aid banks in deciding which units to exclude without having to perform additional expensive characterization. Interestingly, birth weight has been associated with greater collection volumes and could be considered as an initial criterion for the selection of cord blood donors to further increase efficiencies [7]. Although precise minimum thresholds of volumes needed at collection to bank units are typically 40 mL, evidence correlating volume with rates of hematopoietic engraftment remains unclear with regards to minimum volumes. Virtually all cord blood banks report volume collected when their units are listed on international registries.

### 1.2. Total Nucleated Cell Count (TNC)

TNC has emerged as the most commonly reported parameter in addition to volume [3, 9]. Nearly 100% of units captured by recent searches of international cord blood units have a reported TNC count measured prior to freezing. Processing and cryopreservation methods, however, can impact the TNC and these methods vary considerably between banks. Prefreeze TNC counts may not always be predictive of engraftment potential without considering the method of storage. Some experts have suggested a corrective factor to apply to units that are stored following red cell depletion (i.e., buffy coat preparations) or volume reduction methods [10]. Regardless, numerous studies have documented a strong association between TNC counts and engraftment [3]. Historically, in the absence of definitive data, there has been some consensus that the minimal TNC should be 1.5 × 10^7^/kg recipient weight or 1.7 × 10^5^ CD34^+^ cells/kg (see section below) as outcomes are significantly poorer for recipients of cord blood units smaller than this [11] and this is supported by other reports [12, 13]. More recently, some banks have suggested higher thresholds including a recent report from the World Marrow Donor Association which recommends a minimum of 2.0 × 10^7^ TNC/kg or 2.0 × 10^5^ CD34^+^ cells/kg [14]. Jaime-Pérez et al. [8] recently reported on their development of receiver-operator curves and demonstrated that using TNC as a single criterion (0.8 × 10^9^) led to the optimal relationship between sensitivity and specificity with respect to selecting cord blood units (CBUs) for cryopreservation (CD34^+^ = 2 × 10^6^). Minimum TNC doses may vary with the degree of HLA-disparity [6]. For 6/6 and 5/6 HLA matches, a TNC dose of 2 × 10^7^/kg has been identified while for 4/6 matches, a minimum dose of 3 × 10^7^ is recommended [15]. Others have suggested transplant centres (TCs) use a minimum cryopreserved TNC dose of 2.5 × 10^7^/kg for a single unit when considering a 5 of 6 HLA-matched graft, and 5.0 × 10^7^/kg for a single unit when considering a 4 of 6 HLA-matched graft [6, 10, 16]. A minimal threshold of 0.9 × 10^9^ TNC in collected units, therefore, has been proposed as a cost-effective goal for banks to consider when deciding on a threshold for banking [16]. Finally, there is evidence in double cord transplants that a higher TNC count of the dominant cord is associated with more rapid engraftment [17]. Because one cannot predict which cord blood unit will engraft, the authors conclude that both cords must have high counts and they suggest a TNC threshold of 2.0 × 10^7^/kg for each unit of a double graft. Essentially all banks provide TNC information for all listed cord blood units.

### 1.3. Total Mononuclear Cell Counts (MNCs)

Total mononuclear cell counts differ from total nucleated cell counts by excluding polymorphonuclear cells such as neutrophils which are not included in some volume reduction strategies such as the “buffy coat” method of processing. Some groups include MNC counts as part of their characterization although only a minority of banks provide this information in preliminary cord blood searches. Although MNCs may correlate with CD34^+^ cell numbers [18], data relating to rates of hematopoietic engraftment following transplantation are lacking. Decisions to bank based on MNC levels may be most relevant to banks performing specific methods of cell processing and volume reductions.

### 1.4. CD34^+^ Cells

The cell surface antigen CD34 has been most studied in the context of peripheral blood progenitor cell transplantation and has been associated with rates of hematopoietic engraftment in this context [4]. CD34 has also been correlated with rates of engraftment following UCB transplants [5, 13]. CD34 enumeration can be performed prior to storage; however, the technique of CD34 enumeration can yield unreliable results unless performed using single platform flow cytometry in accordance with International Society of Hematopoietic and Graft Engineering (ISHAGE) guidelines [19]. CD34 enumerations are routinely performed by many banks on thawed segments attached to cord blood units although this technique has not been prospectively studied in terms of its correlation with engraftment and remains controversial and under active study. Prefreezing CD34 counts are reported for approximately 10–20% of listed units, represented by a small number of banks which comprise a large cumulative inventory (based on a limited review of actual cord blood searches performed by OneMatch, Canadian Blood Services, in 2010; data not shown). Quantitative insight regarding the impact of CD34 counts on the selection of units by transplant centres remains uncertain. The US Department of Health has recommended a minimum threshold value of 1.2 × 10^6^ CD34^+^ cells per unit although scientific validation of this value remains incomplete. Many banks routinely provide CD34^+^ numbers on listed units although the use of CD34^+^ counts as a method of deciding which units to process and keep in the bank remains largely untested due to cost and time inefficiencies.

### 1.5. Colony-Forming Units (CFU)

Colony-forming units (CFU) can be enumerated by plating mononuclear cells in semisolid methylcellulose in association with appropriate media supplements and hematopoietic growth factors [20]. Colonies emerge in the plastic dishes after 10–14 days and can be enumerated by morphology and classified as granulocytic (CFU-G), monocytic (CFU-M), granulocytic-monocytic (CFU-G/M), erythroid or blast-forming (CFU-E, CFU-B), megakaryocytic (CFU-Meg), and multipotential (CFU-GEMMeg). CFUs have been associated with rates of engraftment in paediatric cord blood transplant [20, 21] but the technique requires a high degree of expertise, is time-consuming, and associated with high interobserver variability. CFU data are not available on typical preliminary reports available to transplant centres following international cord blood searches but are often available on more detailed reports of requested units. The role of CFU in cord blood banking appears most likely to retain relevance in the assessment of attached segments after prospective cord blood units have already been identified using other criteria.

### 1.6. ALDH-hi

Increased expression and enzymatic activity of aldehyde dehydrogenase (ALDH) activity has been associated with increased hematopoietic potential in several research models of transplantation and in the autologous transplant setting [22]. No clinical studies have been performed evaluating this flow cytometric parameter in terms of decision to bank units and no units listed in international searches contain this information. The technique is technically challenging and there are no international guidelines such as the ISHAGE guidelines for CD34 enumeration.

### 1.7. SCID Mouse Repopulating Cells (SRC)

Since human hematopoietic stem cells remain incompletely defined in terms of their immunophenotype, many research teams continue to use a xenogeneic mouse transplant model to demonstrate the existence of hematopoietic stem cells. In essence, enriched cellular populations or sources of hematopoietic cells are transplanted into conditioned immunosuppressed NOD SCID mice [23]. The repopulation of the mouse marrow compartment with human hematopoietic cells that provide longstanding and sustained hematopoiesis proves the existence of hematopoietic stem cells in the starting population. Serial transplants from the transplanted mouse into another mouse strengthen the argument. Needless to say, this assay remains in the realm of scientific biomedical research. The assay is costly and requires a high level of expertise. It is semiquantitative. It may, however, be viewed as the gold standard next to human transplantation for the validation of biomarkers of hematopoiesis [24].

### 1.8. Age of the Unit

Much research has focussed on the age of blood products and the storage lesion [25]. This has mostly centred on red blood cell products and remains the subject of active research. The role of storage time for cord blood units is less clear. Several groups have published reports on the excellent viability of cord blood units that have been stored for many years [26]. In a study by Sumida et al. [27], the average decline in the viability of stem/progenitor cells in umbilical cord blood cryopreserved for up to 7 years was only 0.11% per year. The authors suggest that these data do not support limits to storage time and none of the public banks routinely provide the year of banking in preliminary search reports. Importantly, the current *WMDA International Standards for Unrelated Hematopoietic Stem Cell Donor Registries* [28] do not make any mention of limitations on the storage of cryopreserved cord blood units and the AABB *Standards for Cellular Therapy Product Services* (5th edition) [29] do not directly address the issue of product expiry dates. 

### 1.9. Combining Factors in a “Scoring” System

Recent reports have started to examine the role of combining several factors to optimize selection of cord blood units for transplantation. In a recent paper by Page et al. [30], the hazard ratios for precryopreservation and postcryopreservation graft viabilities for TNC, CD34^+^ cells, CFUs, MNCs, and volume were collected and calculated in terms of their correlation with neutrophil engraftment. The magnitude of the hazard ratios was then used to develop a scoring system that is intriguing. More study is required on the development of scoring systems for selecting cord blood units before a recommendation can be made.

## 2. Current International Guidelines for the Selection of Cord Blood Units for Hematopoietic Transplantation: Impact on the Decision to Bank

It has been suggested that cell dose and number of HLA mismatches interact mutually on engraftment and on other transplant-related outcomes and therefore, a higher cell dose in the graft could partially overcome the negative impact of HLA for each level of HLA disparity. Gluckman et al. [9] determined that cell dose at freezing and the number of HLA mismatches were both associated with rates of neutrophil engraftment. Specifically, they reported that higher numbers of cord blood stem cells reduced the influence of HLA disparities on probabilities and rates of neutrophil engraftment. Accordingly, the authors argue that thresholds for cell dose must consider the degree of HLA disparity that is being considered. The practice of selecting cord units based on a combination of TNC count and HLA disparity is common and is supported by several recent studies and reviews [3, 10]. Similar relationships have been observed for the impact of CD34^+^ cell numbers in cord blood units and rates of engraftment [13] but precise cutoffs with regard to the degree of HLA disparity are less well defined. Interestingly, higher TNC count and CD34^+^ cell numbers are associated with unit dominance in double cord blood transplant and may be used in strategic selection of units in this context [17]. Many banks rely only on TNC counts with regard to decisions to bank and the TNC cut off for banking units continues to increase. Few banks are utilizing CD34 levels, however, in algorithms related to the decision to bank, perhaps owing to the expense of CD34 enumeration on a systematic basis. A more practical approach may be to define a particular birth weight that is predictive of high TNC counts and selectively collect from these units, using a TNC count to decide which units to store. More complete characterization with CD34 levels and CFU levels can then be performed on a prestorage sample to characterize the unit. Public cord blood banks will need to be agile in the development of strategies that provide broad HLA diversity while maintaining an inventory with high stem cell content in order to address the key barriers to increased use of cord blood in hematopoietic stem cell transplantation.

Expert opinions continue to provide guidance regarding international practice with regards to additional parameters that influence cord blood banking [6, 10, 15]. Taken together, accreditation by FACT/Netcord or AABB, good listing practises, reasonable prices and search charges, reliable delivery, current and reliable testing for transmissible diseases, and availability of attached segments on banked units all combine to encourage transplant centres to select cord blood units and increase rates of cord blood unit exportation. It is likely that units listed by banks that have multiple favourable characteristics are more likely to be selected. The evaluation of how transplant centres balance measures of unit quality versus measures of graft adequacy alone, however, remains highly variable and imprecise. In other words, cords with lower TNC counts may remain desirable in the international market if they possess several measures that reflect high quality. Ongoing analysis of economic factors that could influence this delicate balance are ongoing and may be specific to particular jurisdictions.

## 3. Economic Factors in the Use of Cord Blood Units: Implications on Banking Decisions

Detailed information on the importing and exporting of cord blood units was recently reported by Brown et al. [31]. The data was extracted from the World Marrow Donors Association and was augmented with additional information based on utilization rates. Identifying countries with the greatest rates of exportation may provide a starting point for understanding what factors may be contributing to the selection of CBUs on an international scale. Data regarding release and exporting of units relative to inventory for 2008 [31] is presented in Table 1. From this analysis, one can observe a few trends. The largest ratio of released units relative to banked inventory occurs in France (3.0%). The USA, Italy, Germany, and Australia have similar ratios of released units relative to inventory (0.7–0.9%). Many factors may influence the utilization of domestic units including practice patterns of transplant centres within that country, cost of CBUs, and the increased likelihood of finding a match within the same country as the transplant centre due to frequency of common haplotypes for a particular population or ethnicity. To better appreciate whether cords banked in one jurisdiction are preferred by foreign transplant centres, we assessed the ratio of exported units to inventory. France has the highest ratio of exporting units (1% of inventory, [31]), followed by Australia, Germany, and Switzerland. Interestingly, banks with the largest inventories have the lowest ratio of exported units. This may be explained in part by the fact that larger banks are typically older and may have many units with lower cell numbers or lower volumes in their inventory from earlier times. A more updated analysis of banks using data from units that were banked more recently would be insightful. Most banks with high rates of international appeal are performing CD34 counts prior to freezing and reporting these values in the search report. Factors that influence the selection of cord blood units are not clearly defined but include HLA compatibility, cell count (TNC, CD34, or other), origin of the unit (country, bank, accreditation status, credibility), and cost, as described above. It is widely believed that HLA and cell count are the most important factors determining the selection of a cord blood unit. Moreover, two recent papers suggest that the degree of HLA matching is the most dominant selection criteria compared with measures of graft potency when all else is equal [3, 16].

Financial viability of cord blood banking operations is a reality in many jurisdictions. Banking activities have stopped in some cases due to a lack of funds. The exportation of units is a recognized means of recovering costs for cord banks. More study is needed to determine the factors that determine selection of cord blood units in terms of economic viability of cord blood banks. Evidence presented at the International Cord Blood Symposium 2012 illustrates an international trend to banking cords with higher TNC largely due to economic forces. Data from The National Marrow Donor Program (NMDP) suggests that only 1.0% of listed cords are selected for transplant. Moreover, cords banked with a TNC between 0.9 and 1.24 × 10^9^ represented less than 0.6% of the total units selected by transplant centres with no difference in the proportion of units derived from dominant and nondominant ethnic donors and controlling for transplant recipient variables such as ethnicity and weight. These preliminary data suggest that ethnicity does not account for the selection of smaller units. Moreover, current utilization data reveal a median TNC of units shipped for transplant of 1.88 × 10^9^ (NMDP, presentation at San Francisco, June 2012). Modelling done by the NMDP suggests a “crossover point” of 1.75 × 10^9^ at which point it is more cost effective to recruit adult registry donors rather than bank cords with a TNC below this threshold (unpublished data).

Review of the Canadian Datacord data for the past two years provides a summary for UCB transplants in Canadian recipients (see Table 2). The data is further broken down to reveal average TNC per individual CBU within double cord transplants. These confirm the recent trends supporting higher TNC doses for adult recipients and the increasing use of double cord blood transplantation as a strategy to overcome limiting cell doses.

## 4. Summary: Striking the Balance on How to Decide Which Units to Take into Inventory

By their nature CBUs are small with lower absolute numbers of progenitor cells compared to peripheral blood or marrow donations. With increasing use of cord blood in adult transplantation, there is economic pressure to bank only the largest units (over 1.75 × 10^9^ TNC) with the subsequent potential for reduced HLA diversity within the bank. There appears to be a continuum along which the balance of HLA disparity and TNC dose reciprocates and banks must find an appropriate balance along this continuum that meets the needs of their particular mandate. Cord blood banks must also focus on additional factors to ensure that the cords taken into the bank are of the highest quality and thus remain desirable to transplant centres and their patients. Accreditation and compliance with nationally and internationally recognized regulatory bodies ensure that all aspects of recruitment, donor screening, collection and transport, processing, testing, freezing, storage, and distribution are standardized and meet international thresholds of quality. The availability of attached segments and postproduction and/or postthaw testing allows for confirmation of identity and assurance of potency and complement tools utilized to make decisions regarding whether to bank units or not. Reliable and reproducible CD34^+^ and CFU assay testing must be achieved and ultimately standardized within and eventually between banks. Additional features such as high-resolution HLA typing of all alleles, KIR ligand testing, and testing maternal samples to match for noninherited maternal HLA antigens (NIMA) may also improve transplant outcomes and warrant consideration. As the list of tests with “added value” to transplant centres increases, cord blood banks must balance the costs associated with these tests with strategies that allows them to be more selective with regards to criteria used to select cord blood units that are banked. Cord blood banks offering superior quality products to transplant centres and their patients will render them more financially viable in the long term and better able to balance the issue of selecting additional cords of superior quality into their inventory. Striking the balance will be the key to successful cord blood banking and will require ongoing evaluation.

## 5. Initiating a National Cord Blood Bank in Canada: A Current Case of Putting the Evidence into Practice

Canadian Blood Services is embarking on the establishment of a national public cord blood bank in Canada and has the opportunity to put into practice the issues discussed in this paper. The main goals of the Canadian initiative are to provide a bank with broad HLA diversity that includes significant representation of ethnic minority groups and Aboriginal peoples. At the same time, cord blood units with high-graft potency are desired, knowing the demands of transplant centres in Canada and worldwide. Cord blood units that exceed a minimum volume of 40 mL will be tested for TNC count and screened for banking eligibility. Units with at least 1.5 × 10^9^ TNC will be considered for banking and will undergo more extensive testing. Data regarding ethnicity will be captured during a pilot phase to determine the extent that donor ethnicity influences TNC counts. This will assist the bank in deciding whether different TNC thresholds are needed in the future to maintain an inventory that is representative of the ethnic mix required to increase transplant opportunities for patients in need. Additional strategies may be required to avoid excessively high collection to banking ratios. It may be necessary in the future to embrace strategies of approaching mothers of infants with birth weights above a particular threshold but there is insufficient evidence at this time to use this strategy from the beginning and the logistics of such a strategy may pose challenges. An informing system of feedback is needed to ensure iterative improvements to the process of cord blood banking and to ensure that the precious allocated resources yield the most beneficial clinical outcomes for patients in need of hematopoietic stem cell transplantation. 

## Figures and Tables

**Table 1:** Analysis of cord blood units released and exported from cord blood banks around the world.

Country	Inventory (2008)	Released (2008)	Exported
France	7051	212 (3.0%)	74 (1.0%)
Australia	20044	178 (0.9%)	133 (0.7%)
Germany	18557	127 (0.7%)	110 (0.6%)
Switzerland	2212	13 (0.6%)	13 (0.6%)
Belgium	14533	74 (0.5%)	68 (0.5%)
Italy	17503	141 (0.8%)	94 (0.5%)
USA	154749	1428 (0.9%)	512 (0.3%)
UK	10589	47 (0.4%)	37 (0.3%)
Spain	35802	179 (0.5%)	123 (0.3%)

**Table 2:** TNC counts on umbilical cord blood units utilized in Canada 2010-2011.

	Adults 2010	Adults 2011	Pediatrics 2010	Pediatrics 2011
Mean TNC × 10^9^ (range), single CBU transplants	2.7 (1.53–5.47)	2.70 (1.22–3.65)	1.77 (0.128–2.94)	1.88 (0.95–3.68)
Mean TNC × 10^9^ (range) per unit in double CBU transplants	1.69 (1.09–3.25)	1.35 (1.02–2.13)	1.21 (0.03–2.50)	1.86 (0.79–2.62)
Mean total TNC × 10^9^ (range), double CBU transplants	4.60 (2.79–6.33)	2.70 (2.14–3.25)	3.20 (2.56–3.58)	4.04 (3.13–5.28)
